# Transcranial Doppler Ultrasound Velocity Measurements in Children With Sickle Cell Disease in Kenya

**DOI:** 10.1002/jha2.70312

**Published:** 2026-05-19

**Authors:** Catherine Mwalimu, Oscar Odhiambo, Elaine Mashisia, Teresa Mwanja, Allan Ade, Maria Vittoria De Vita, Katherine Oyieke, Pauline Samia, Fenella Kirkham, Samson Gwer

**Affiliations:** ^1^ Department of Paediatrics and Child Health Aga Khan University Hospital Nairobi Kenya; ^2^ Department of Paediatrics and Child Health Kitui County Referral Hospital Kitui Kenya; ^3^ Ubuntu Neurology Nairobi Kenya; ^4^ Department of Neurology and Neurophysiology Gertrude's Children's Hospital Nairobi Kenya; ^5^ Department of Paediatrics and Child Health Ruaraka Uhai Neema Hospital Nairobi Kenya; ^6^ Developmental Neurosciences Department University College London London UK; ^7^ School of Health Sciences Kenyatta University Nairobi Kenya

**Keywords:** cerebral blood flow velocity, sickle cell disease, stroke prevention, transcranial Doppler

## Abstract

**Background:**

Sickle cell disease (SCD) is the most common inherited haemoglobinopathy and poses a high public health burden in Sub‐Saharan Africa. Children with sickle cell anaemia are at a highrisk of ischemic stroke, and transcranial Doppler (TCD) ultrasonography helps identify those at highest risk for primary stroke prevention.

**Objective:**

To determine the prevalence of abnormal cerebral blood flow velocity (CBFV) in children with SCD and describe CBFV in children without SCD.

**Methods:**

We conducted a two‐component study comprising a retrospective review and a cross‐sectional assessment. TCD measurements from 143 children with confirmed SCD aged 2–16 years were reviewed. In a cross‐sectional arm, children without SCD aged 3–11 years (*n* = 17) underwent standardized middle cerebral artery (MCA) TCD assessment. TCD velocities were categorized using standard criteria and analysed descriptively.

**Results:**

Among 143 children screened, all of whom were receiving hydroxyurea, 1.4% had high TCD velocities (≥ 200 cm/s), while 4.9% had low velocities (< 50 cm/s). TCD velocities were not associated with demographic/clinical characteristics or haemoglobin levels. Symmetric MCA flows with wide depth and velocity range variability were observed. In children without SCD, all MCA velocities were within the normal range (50–149 cm/s), showed right–left symmetry, declined with age, and were broadly similar by gender.

**Conclusion:**

The prevalence of abnormal TCD velocities was low in children with SCD receiving universal hydroxyurea. In children without SCD, MCA velocities were normal and declined with age, providing local reference values. The observed variability in CBFV supports continued annual TCD screening and repeat scans to confirm stroke‐risk classification.

**Trial Registration:**

The authors have confirmed clinical trial registration is not needed for this submission.

## 1 Introduction

### 1.1 Background

Sickle cell disease (SCD) is the most common inherited haemoglobinopathy, affecting over 300,000 children born annually worldwide [1, 2]. Approximately 75% of cases occur in Sub‐Saharan Africa (SSA), where mortality rates for children under five with SCD range from 50% to 80% [1, 3].

SCD results from a point mutation in the beta‐globin gene that causes haemoglobin polymerization under low oxygen tension, leading to red cell sickling, haemolysis and microvascular obstruction [1]. These processes contribute to end‐organ injury, including cerebrovascular disease and stroke, one of the most severe complications in children with sickle cell anaemia (SCA) [3, 4]. Studies in Uganda and Nigeria have reported stroke prevalences of 6.8% and 5.4%, respectively, in children with SCA, with recurrence risk as high as 61.5% [5, 6]. Most children with overt stroke have underlying large‐vessel stenosis or occlusion, affecting the internal carotid and middle cerebral arteries [7].

Transcranial Doppler (TCD) ultrasonography is essential for assessing stroke risk in children with SCD, with current guidelines recommending annual screening for children aged 2–16 years [8]. Cerebrovascular flow velocity (CBFV) > 200 cm/s indicates a significantly increased risk of stroke [9]. High‐risk children are generally managed with regular prophylactic blood transfusions as the primary preventive strategy [10].

While TCD values have been established in various populations, studies in Africa and the UK report considerable variability in the prevalence of cerebrovascular abnormalities among children with SCD. Studies in Nigeria, Egypt and Tanzania have reported abnormal TCD velocities (≥ 200 cm/s) in 0.5%–19.7% of children and conditional velocities (170–199 cm/s) in 5%–20% [9, 10, 11, 12]. Differences in TCD velocity distributions across populations raise concerns about how well existing thresholds and risk patterns generalize to African clinical settings [13]. This population‐level variability extends beyond Africa; studies in Kuwaiti and peninsular Arab children with SCD report uniformly low TCD velocities with no patient exceeding 200 cm/s, which is attributed to high foetal haemoglobin levels associated with the Arab‐Indian haplotype [14, 15].

Kenya has a substantial burden of SCD, but local data on TCD velocities in Kenyan children with SCD and normative TCD velocity values in children without SCD remain limited. We conducted this study to determine the prevalence of abnormal TCD measurements in children with SCD and to determine TCD velocity measurements in children without SCD in Kenya. The findings aim to provide locally relevant data to improve stroke risk assessment and inform priority interventions for this population.

See Supporting Information for the list of abbreviations.

## 2 Methods

### 2.1 Study Design

This study had two components: a retrospective observational arm and a cross‐sectional arm. The retrospective arm examined TCD measurements in children with SCD at Gertrude's Children's Hospital (GCH) and Ruaraka Uhai Neema Hospital (RUNH), Nairobi, Kenya. The cross‐sectional arm measured TCD cerebral blood flow velocity (CBFV) in children without SCD at GCH.

### 2.2 Study Setting

The study was conducted in Nairobi, Kenya, at GCH and RUNH. Cross‐sectional recruitment and TCD assessments for children without SCD were performed at GCH (additional setting details are provided in the Supporting Information).

### 2.3 Study Population

The retrospective cohort consisted of children aged 2–16 years with SCD confirmed by haemoglobin electrophoresis or genetic testing, who underwent TCD assessment at the haematology and neurology clinics between July 2021 and December 2024. Children were excluded if they had a history of neurosurgical intervention, significant head trauma, or known cardiovascular disease. TCD assessments in the retrospective arm were performed during routine outpatient clinic visits, which typically represent a steady clinical state. However, formal confirmation of steady state at the time of each scan was not possible from retrospective records alone, and the possibility of subclinical complications at the time of assessment could not be excluded.

The cross‐sectional cohort comprised clinically well children aged 3–11 years attending the Orthopaedic or Well‐Baby clinics at GCH. Eligibility criteria included normal growth for age (per WHO growth charts), normal neurodevelopment (Molteno Developmental Scale), and a haemoglobin level of 11.0–14.5 g/dL measured by HemoCue at the time of assessment. Children were excluded if they had any of the following: acute illness, fever or suspected CNS infection; chronic transfusion therapy or prior bone marrow transplant; asthma or other chronic respiratory disease; a history of stroke or neurodevelopmental abnormality; prior neurosurgery or significant head trauma; or known cardiovascular disease.

### 2.4 Data Management and Analysis

We included all eligible children with SCD assessed during the study period (July 2021–December 2024). For the cross‐sectional arm, we aimed to recruit approximately 20 children without SCD. Sample size considerations are detailed in the Supporting Information.

### 2.5 Data Collection

For the retrospective arm, TCD and clinical data were extracted from digital records at GCH and RUNH. For the cross‐sectional arm, parents/guardians completed a structured medical questionnaire and participants underwent a focused clinical assessment; haemoglobin was measured at the time of TCD using HemoCue.

TCD was performed by trained neurophysiologists using a non‐imaging pulsed‐wave spectral Doppler system with a 2‐MHz probe (DWL Multi‐Dop T, Doppler Electronic Systems, Sipplingen, Germany) via trans‐temporal insonation of the middle cerebral artery (MCA). Flow velocities were derived from pulsed‐wave spectral Doppler waveforms; colour‐flow imaging was not used for velocity quantification. The primary velocity parameter was MCA mean peak flow velocity; where bilateral measurements were recorded, the higher value was used for velocity categorization. Full technical acquisition details are provided in the Supporting Information.

### 2.6 TCD Velocity Classification

Velocities were categorized as: low (< 50 cm/s), normal (50–149 cm/s), slightly elevated (150–169 cm/s), conditional risk (170–199 cm/s) and abnormal (≥ 200 cm/s), based on MCA mean peak flow velocity. This classification was adapted from a validated Tanzanian paediatric study [12] (see Supporting Information for classification rationale).

### 2.7 Data Analysis

Data analysis was conducted using Stata version 17. Categorical variables were summarized as frequencies and percentages; continuous variables as median and range. The prevalence of abnormal TCD velocities was calculated as a proportion of the total sample. Fisher's exact test compared demographic and clinical characteristics across TCD velocity categories. Wilcoxon rank‐sum tests assessed side‐to‐side differences by age. Statistical significance was set at 5%. Analyses for children without SCD were primarily descriptive, given the small sample size. Additional statistical details are provided in the Supporting Information.

### 2.8 Ethical Considerations

Ethical approval was obtained from the Research Ethics Committee of Gertrude's Children's Hospital and the Aga Khan University, Nairobi Institutional Scientific and Ethics Review Committee (ISERC). A waiver of consent was granted for the retrospective review; written informed consent (and assent for children aged ≥ 7 years) was obtained for the cross‐sectional arm. Additional methodological details are provided in the Supporting Information.

## 3 Results

A total of 143 children with SCD were included in the retrospective arm (median age 7.5 years [2–16]; 54.5% male, *n* = 78). Most were Luo (77.6%, *n* = 111). Anthropometry was available for 135 children; most had normal weight‐for‐age (*n* = 95) (Table 1). Seventeen children were included in the normal arm of the study. They had a median age of 6 (IQR 5–9.5) and 58.8% (*n* = 10) were males. Their clinical characteristics are provided alongside those of SCD children in Table 1.

**TABLE 1:** Baseline characteristics of study participants.

**Characteristic**	**Children with SCD (*n* = 143)**	**Children without SCD (*n* = 17)**
Demographics		
Gender, *n* (%)		
Male	78 (54.5)	10 (58.8)
Female	65 (45.5)	7 (41.2)
Age		
Median (IQR), years	7.5 (2–16)	6 (5–9.5)
Age category, *n* (%)		
2–5 years	51 (35.7)	5 (29.4)
6–10 years	55 (38.5)	9 (52.9)
11–16 years	37 (25.9)	3 (17.6)
Ethnicity, *n* (%)		
Luo	111 (77.6)	4 (23.5)
Kikuyu	1 (0.7)	8 (47.1)
Luhya	16 (11.2)	0 (0)
Kamba	0 (0)	2 (11.8)
Somali	0 (0)	2 (11.8)
Digo	0 (0)	1 (5.9)
Pokomo	3 (2.1)	0 (0)
Kisii	2 (1.4)	0 (0)
Unknown	10 (7.0)	0 (0)
Anthropometric measurements		
Weight for age, *n* (%)		
Underweight (< 5th percentile)	19 (14.0)	1 (5.9)
Normal (5th–95th percentile)	95 (70.4)	13 (76.5)
Overweight (> 95th percentile)	21 (15.6)	3 (17.6)
Medical history		
Previous hospital admissions, *n* (%)	104/132 (78.8)	5 (29.4)
Chronic medical conditions, *n* (%)	56/132 (42.4)	3 (17.6)
Allergic rhinitis/ allergic conjuctivitis		2 (11.8)
Retro‐viral disease(RVD)		1(5.9)
Developmental delay, *n* (%)	6/132 (4.5)	1 (5.9)

Clinical information was available for 132 children with SCD. Most (79%, *n* = 104) had prior admissions, mainly for sickle cell crises (painful crises: 59%, *n* = 61). Comorbidities were reported in 39% (*n* = 56), most commonly respiratory conditions. Neurological complications occurred in 9% (*n* = 12), predominantly stroke related and 46% (*n* = 48) had received transfusions. All children were receiving hydroxyurea at TCD assessment, and about two‐thirds had haemoglobin < 10 g/dL (*n* = 71). Characteristics are summarized in Table 2.

**TABLE 2:** Clinical characteristics of children with sickle cell disease.

**Clinical characteristics**	**Frequency**	**Percent**
History of previous admissions	104	78.8
Reason for admission (*N* = 104)		
Painful crisis	61	58.7
Severe anaemia	27	26.0
Acute chest syndrome	9	8.7
Pneumonia	17	16.3
Adenoid hypertrophy	11	10.6
Severe malaria	10	9.6
Tonsillitis	7	6.7
Rhinitis	4	3.8
Otitis media	3	2.9
Bronchiolitis	2	1.9
Others^a^	5	4.8
Asthma	14	9.8
Background history of neurological conditions		
Stroke	8	66.7
Epilepsy	3	25
Headache	2	16.7
Developmental delay	6	4.5
History of blood transfusion	48	46.2

^a^ Choanal atresia (1), cholelithiasis (1), meningitis (1), febrile seizures (1).

### 3.1 CBFV Measurements

Only two (1.4%) children had abnormal velocities; four (2.8%) had conditional velocities and six (4.9%) had low TCD velocities.

TCD measurements showed right–left MCA symmetry, with similar median depth (44 mm) and comparable median mean velocities (right 114.5 cm/s [IQR 99–129] vs. left 113.5 cm/s [IQR 97.5–128]). The median absolute side‐to‐side difference was 12 cm/s (10.6%), as shown in Table 3.

**TABLE 3:** Cerebral blood flow velocity (CBFV) measurements.

**Measurements**	**Children with SCD**	**Children without SCD**
Right MCA peak systolic velocity (cm/s), median (IQR)	165.5 (139–185)	
Right MCA end diastolic velocity (cm/sec), median (IQR)	75 (60–90)	
Right MCA mean peak flow velocity (cm/sec), median (IQR)	114.5 (99–129)	102 (85–107)
Left MCA peak systolic velocity (cm/sec)	165.5 (142–187.5)	
Left MCA end diastolic velocity (cm/sec)	75 (60–88)	
Left MCA mean peak flow velocity (cm/sec)	113.5 (97.5–128)	100 (90–112)
MCA mean flow velocity absolute differences	12.0 (5, 6, 7, 8, 9, 10, 11, 12, 13, 14, 15, 16, 17, 18, 19)	14 (7–29)
MCA mean peak flow velocity percentage differences	10.6 (4.9–18.7)	12 (6–31)

There were no significant differences in depth, peak systolic, end‐diastolic, or mean flow velocities between sides across age brackets (all *p* > 0.05).

In children without SCD (*n* = 17), all velocities were within the normal range (50–149 cm/s), with comparable right–left depths and velocities. Velocities declined with age, peaking in the 3–5‐year group, and distributions were similar by gender, as shown in Table 3.

### 3.2 CBFv Versus Clinical Characteristics

All children with abnormal TCD had prior SCD‐related admissions and low haemoglobin; however, there was no significant difference across TCD categories, as presented in Table 4.

**TABLE 4:** CBFv examined across different clinical characteristics.

**Characteristics**	**Normal *n* (%)**	**Low *n* (%)**	**Slightly elevated *n* (%)**	**Conditional risk *n* (%)**	**Abnormal *n* (%)**	***p* value**
Gender (*N* = 143)						0.403
Male	65(53.3)	3(42.9)	7(87.5)	2(50.0)	1(50.0)	
Female	57(46.7)	4(57.1)	1(12.5)	2(50.0)	1(50.0)	
Age (*N* = 143)						0.173
2–5 years	59(48.4)	3(42.9)	6(75.0)	0	1(50.0)	
6–10 years	41(33.6)	2(28.6)	2(25.0)	4(100)	1(50.0)	
11–16 years	22(18.0)	2(28.6)	0	0	0	
Weight for age (*N* = 135)						0.286
Underweight	13(11.3)	3(42.9)	1(12.5)	2(66.7)	0	
Normal (5th–95th percentile)	85(73.9)	4(57.1)	3(37.5)	1(33.3)	2(100)	
Overweight (> 95th percentile)	17(14.8)	0	4(50.0)	0	0	
Ethnicity (*N* = 143)						0.996
Kikuyu	1(0.8)	0	0	0	0	
Kisii	2(1.6)	0	0	0	0	
Luhya	14(11.5)	1(14.3)	1(12.5)	0	0	
Luo	92(75.4)	6(85.7)	7(87.5)	4(100)	2(100)	
Pokomo	3(2.5)	0	0	0	0	
Unknown	10(8.2)	0	0	0	0	
Previous admissions						0.918
Yes	89(78.8)	5(83.3)	6(75.0)	2(66.7)	2(100)	
Admission due to SCD‐related crisis						0.863
Yes	70(57.4)	4(57.1)	6(75.0)	2(50.0)	1(50.0)	
Admission due to other medical conditions						0.313
Yes	45(39.8)	5(83.3)	4(50.0)	1(33.3)	1(50.0)	
History of neurological conditions (*N* = 131)						0.288
Yes	9(8.0)	1(16.7)	1(12.5)	0	1(50.0)	
History of blood transfusion (*N* = 104)						0.354
Yes	40(44.9)	4(80.0)	2(28.6)	1(100.0)	1(50.0)	
History of developmental delay(*N* = 131)						0.901
Yes	6(5.3)	0	0	0	0	
Haemoglobin level						0.419
Normal	34(36.6)	0	3(37.5)	0	0	
Low (< 10 g/dL)	59(63.4)	3(100)	5(62.5)	2(100)	2(100)	

### 3.3 Stroke Occurrence and TCD Risk Classification

Eight children had a history of stroke: seven (6%) had normal TCD measurements and one had an abnormal TCD measurement. All children with documented stroke had a history of SCD‐related admissions.

## 4 Discussion

This study examined TCD ultrasound velocities in 143 Kenyan children with SCD aged 2–16 years. We found a low prevalence of abnormal cerebral blood flow velocities, with 1.4% (95% CI: 0.2%–5.0%) of children having velocities ≥ 200 cm/s. Conditional velocities (170–199 cm/s) occurred in 2.8%, with a further 5.6% showing slightly elevated velocities (150–169 cm/s). Low velocities (< 50 cm/s) were observed in 4.9%.

This prevalence is lower than that reported in West African studies. In Nigerian paediatric SCD cohorts, abnormal TCD velocities have typically been in the 4.5%–9.3% range, while conditional velocities have ranged from about 13.6% to19.7% [10, 13, 16]. Both abnormal and conditional velocities were higher than in our cohort.

However, our findings are consistent with reports from East Africa (Kenya, Tanzania) and from other African settings, including Egypt and Central Africa, where abnormal velocities are uncommon and conditional velocities are more frequently observed than abnormal velocities [9, 11, 12, 17]. Similarly low rates have been reported in Arab SCD cohorts, where overt stroke occurred in only one of 59 patients, underscoring the influence of genetic background on cerebrovascular risk [14].

Median MCA velocities were similar between sides (right: 114.5 cm/s; left: 113.5 cm/s) with a median absolute difference of 12 cm/s, showing no significant right–left differences across age groups. These findings align with Congolese and Sudanese SCD cohorts reporting comparable MCA mean velocities with minimal right–left differences [18, 19]. Median MCA insonation depths (44 mm bilaterally) were within paediatric reference ranges [20]. However, the right‐sided depth values showed a wide range (0–128 mm), suggesting occasional variability in recorded measurements. This variability shows the need for repeat TCDs in outliers to confirm risk categorization.

Most children were aged 6–10 years (38.5%), and all four children with conditional velocities and one of the two children with abnormal velocities were within this age range. Similar age distributions of elevated velocities have been reported in Brazil, Tanzania and Kilifi, Kenya [11, 21, 22]. No sex differences in velocity category were detected, which is similar to previous studies indicating minimal or inconsistent gender effects on paediatric TCD velocities [11, 13, 21, 22].

Our study observed a high burden of SCD‐related complications (frequent prior admissions, blood transfusions and neurological complications including prior stroke in 9.2%). Anaemia (Hb < 10 g/dl) was prevalent in about two‐thirds of children and present in all children with conditional velocities and both with abnormal velocities. However, haemoglobin status did not differ significantly across TCD velocity categories, likely due to the small number of children with elevated TCD velocities. These findings contrast with multiple previous studies from Brazil, Italy and Tanzania, which consistently showed an inverse relationship between haemoglobin levels and cerebral blood flow velocities [21, 22, 23].

Stroke was observed in 5.6% of children with normal TCD velocities and in one of the two children with abnormal velocities. The association between TCD risk classification and stroke history was borderline and not statistically significant, likely reflecting the small number of children with elevated velocities. All eight children with a history of stroke were on hydroxyurea at the time of TCD assessment, with doses available for five ranging from 600 to 1000 mg daily. However, treatment duration could not be reliably determined from the retrospective records. Seven of the eight had normal TCD velocities despite their stroke history, suggesting that hydroxyurea may have contributed to cerebrovascular stabilization in the majority. The two children with abnormal velocities, a 9‐year‐old boy with prior stroke and haemoglobin of 7.9 g/dL, and a 4‐year‐old girl with haemoglobin of 7.2 g/dL, both had low haemoglobin at assessment, suggesting that anaemia may reduce the cerebrovascular protective effect of hydroxyurea in some children. These findings support continued annual TCD screening despite universal hydroxyurea use.

To support interpretation of these findings, TCD measurements were also assessed in 17 clinically well Kenyan children without SCD aged 3–11 years (median age 6years). All had MCA velocities within the normal risk category (50–149 cm/s). Median right MCA mean peak velocity was 102 cm/s (IQR 85–107) and median left MCA was 100 cm/s (IQR 90–112), suggesting right–left symmetry. These values are comparable to large African paediatric datasets reporting MCA mean flow velocities typically in the range of approximately 74–88 cm/s across childhood, with higher velocities observed in early and mid‐childhood [24].

Median MCA insonation depths were 44 mm bilaterally, which is within reported paediatric reference ranges used in TCD practice [20]. Some side‐to‐side variability was present, but all children remained within the normal category, consistent with previous studies reporting small to moderate asymmetry (around 11%) in healthy children [24].

By age, velocities were lower in older children than in younger children. This age pattern is consistent with published African paediatric data showing higher MCA mean flow velocities in early and mid‑childhood, followed by lower velocities in older children [17].

Although the sample size in the cross‐sectional arm was relatively small, these data provide locally derived reference values showing that age‐related patterns, side‐to‐side variability, and absolute MCA velocity ranges in children without SCD are consistent with published African paediatric datasets, supporting the use of standard TCD interpretation criteria in Kenya.

This study has important clinical implications for the management of SCD in Kenya and similar resource‐limited settings. The low prevalence of abnormal TCD velocities among children receiving universal hydroxyurea supports its continued use for cerebrovascular protection and primary stroke prevention. However, the occurrence of stroke in children with normal TCD velocities shows that TCD alone does not eliminate stroke risk, making continued annual screening essential. The locally derived reference values from healthy Kenyan children also provide a more practical and relevant guide for interpreting paediatric TCD findings in this setting, reducing reliance on data from different populations. Overall, these findings support the integration of routine TCD screening into standard SCD care protocols in Kenya.

### 4.1 Strengths and Limitations

#### 4.1.1 Strengths

This study provides locally relevant Kenyan TCD data from two clinical sites in a cohort where hydroxyurea use was universal, making the findings applicable to current practice. The inclusion of a cross‑sectional arm of clinically well children without SCD provides locally derived measurements for MCA depth and velocities in Kenyan children.

#### 4.1.2 Limitations

Very few children had abnormal velocities, which reduces statistical power to detect associations. The small cross‑sectional sample size reduces generalizability to the wider Kenyan paediatric population.

## 5 Conclusion

This study found a low prevalence of abnormal TCD velocities (1.4%) among 143 Kenyan children with SCD receiving hydroxyurea, supporting continued annual screening to identify high‐risk cases despite treatment. Symmetric MCA flows with wide variability in depths and velocities highlight the need for repeat TCD in children at ongoing risk to confirm risk category. In clinically well Kenyan children without SCD, MCA velocities were normal and declined with age, providing local reference data for interpreting paediatric TCD findings.

## 6 Recommendations

Children with SCD should continue annual TCD screening despite hydroxyurea use to identify those needing additional stroke‐prevention interventions. Repeat TCD should be performed after elevated velocities or technically suboptimal measurements to confirm risk category and guide risk‐based follow‐up and management.

## Author Contributions

C.M. conceptualized the study, curated the data, developed the methodology, managed the project resources and drafted the original manuscript. O.O., T.M., A.A. and M.V.D.V. contributed to data curation. E.M. contributed to the study conceptualization and data curation. K.O. and P.S. provided  supervision. F.K. contributed to the review of the manuscript. S.G. contributed to principal supervision, review of the manuscript and validation. All authors reviewed, revised, and approved the final manuscript.

## Funding

The authors have nothing to report.

## Ethics Statement

Ethical approval was obtained from the Research Ethics Committee of Gertrude's Children's Hospital and the Aga Khan University, Nairobi Institutional Scientific and Ethics Review Committee (ISERC). Permission to access records was granted by Ruaraka Uhai Neema Hospital, and a NACOSTI research permit was obtained.

## Consent

A waiver of consent was approved for the retrospective review; written informed consent (and assent where applicable) was obtained for the cross‐sectional arm.

## Conflicts of Interest

The authors declare no conflicts of interest.

## Supporting information




**Supporting file 1**: jha270312‐sup‐0001‐SupMat

## Data Availability

The datasets used and analysed during the current study are available from the corresponding author on reasonable request, subject to approval by the relevant ethics committees/institutions.
