# Correction to “Transcranial Doppler Ultrasound Velocity Measurements in Children With Sickle Cell Disease in Kenya”

**DOI:** 10.1002/jha2.70380

**Published:** 2026-08-07

Mwalimu C., Odhiambo O., Mashisia E., Mwanja T., Ade A., De Vita M. V., Mutua D. K., Oyieke K., Samia P., Kirkham F., Gwer S. Transcranial Doppler Ultrasound Velocity Measurements in Children With Sickle Cell Disease in Kenya. *eJHaem*. 2026;7:e70312. https://doi.org/10.1002/jha2.70312.

Dr. Doreen Karimi Mutua was inadvertently omitted from the author list at the time of submission. She made substantial contributions to the study, including patient identification and recruitment; clinical management and interpretation within the sickle cell disease programme; participation in study design and conceptual discussions; and critical review of the manuscript prior to submission. She meets ICMJE criteria for authorship. The correct and complete author list should read: Catherine Mwalimu, Oscar Odhiambo, Elaine Mashisia, Teresa Mwanja, Allan Ade, Maria Vittoria De Vita, Doreen Karimi Mutua, Fenella Kirkham, Katherine Oyieke, Pauline Samia, Samson Gwer. Dr. Doreen Karimi Mutua (MBChB, M.Med (Paed), Dip. Haematology/Oncology; Gertrude's Children's Hospital, Nairobi, Kenya; tessmutua@gmail.com) should appear as the 7th author, between Maria Vittoria De Vita and Fenella Kirkham. All other authors and their order remain unchanged.

We apologize for this error.

