# Supplementary material for: Transcranial Doppler Ultrasound Velocity Measurements in Children With Sickle Cell Disease in Kenya
Source: EJHaem. 2026 May 19;7(3):e70312. doi: 10.1002/jha2.70312 (PMC13185219; doi:10.1002/jha2.70312)
Supplement: Supplementary file 1 — Supporting file 1: jha270312‐sup‐0001‐SupMat [file JHA2-7-e70312-s001.docx]

**Supplementary Methods**

**S1: Study setting**

The study was conducted at two hospitals: Gertrude’s Children Hospital (GCH) and Ruaraka Uhai Neema Hospital (RUNH) in Nairobi, Kenya. Gertrude's Children's Hospital in Nairobi, Kenya, is a tertiary hospital that caters to children and youths up to 21 years old. It serves a diverse population, including residents and referrals from outside Nairobi and the wider East and Central African region. The hospital has a network of 14 clinics that connect to a 104-bed inpatient facility and a 14-bed ICU for admitted patients.

The Pediatric Hematology Clinic at Gertrude's Children's Hospital provides specialized care for children and adolescents with blood disorders, including sickle cell disease (SCD). Led by a dedicated pediatric hematology specialist, the clinic holds a bi-weekly hematology clinic. A team of experienced pediatricians, clinicians, nurses, and pharmacists support the specialist. The hospital has two Transcranial Doppler (TCD) machines: a multi-Dop T system (DWL Doppler) with 2-MHz pulsed-wave Doppler, that are used at both GCH and RUNH. Additionally, the hospital pharmacy stocks hydroxyurea, a medication for SCA, which patients can obtain through insurance or by paying out of pocket.

The neurology department operates outpatient services on all the weekdays, supported by six pediatric neurologists, four neurology trainees, three neurophysiologists, pharmacists, and nurses. The team has experienced specialists who possess the requisite skills to conduct TCD scans.

Ruaraka Uhai Neema Hospital (RUNH) is a faith-based healthcare facility founded in 2008 in Kenya. It is a secondary-level facility that serves a catchment population that is predominantly from the Eastern part of Nairobi: a low and middle income setting. The hospital sees an average of 10,000 patients per month, making it an essential resource for clinics in the low-resource setting. It hosts a clinic for patients with sickle cell disease, which runs once a week from 8 am to 4 pm. Children with Sickle cell disease are seen by a pediatrician and clinical officers. They have access to a TCD machine through an outreach relationship with GCH. The clinic has access to hydroxyurea, which is provided to patients at no cost through sponsorship arrangements.

We retrospectively extracted TCD and clinical data for eligible children with confirmed SCD from digital records at GCH and RUNH (July 2021–December 2024). The study team, in collaboration with neurophysiologists, reviewed these records to verify eligibility criteria, including age range, confirmation of SCD diagnosis, and absence of exclusion criteria.

Cross-sectional recruitment and TCD assessments for children without SCD were limited to GCH due to logistical considerations, including availability of trained personnel and the costs associated with conducting the study at multiple sites. TCD assessments at GCH were performed by trained neurophysiologists using a 2-MHz pulsed-wave Doppler system (DWL).

**S2: Data management and analysis**

Sample size considerations were based on expected differences in the prevalence of abnormal TCD velocities reported in prior studies. In a Nigerian cross-sectional study of 145 children with sickle cell disease, 4.7% of children with HbSS were reported to have abnormal TCD velocities (≥200 cm/s). In contrast, the STOP extended follow-up study, found 22.2% of TCD velocities to be abnormal. Working with an expected proportion of 4% of abnormal TCD observations compared to the STOP observation of 22%, 90% power, and 5% significant level, it was desirable to make observations in at least 35 individuals. In consideration of available data, we made observations on at least 105 individuals, being all the eligible children assessed during the study period and three times the preferred sample size, aligning with previous studies, and to improve the precision of our estimates.

For the cross-sectional arm, we aimed to recruit 20 children for verification of normal TCD values in this setting.

**S3: Data Collection**

For the cross-sectional arm, parents/guardians completed a structured medical questionnaire covering medical history (including neurological, cardiovascular and respiratory conditions), prior transfusion history, admissions, head trauma, and developmental milestones. Participants underwent a focused clinical assessment including measurement of vital signs (blood pressure and oxygen saturation), anthropometry and targeted general and neurological examination. Hemoglobin was measured at the time of the TCD assessment using HemoCue from a finger-prick sample using standard aseptic technique.

TCD assessments were performed by trained neurophysiologists using a non-imaging pulsed-wave spectral Doppler system with a 2-MHz probe (DWL Multi-Dop T, Doppler Electronic Systems, Sipplingen, Germany), consistent with the standard approach for paediatric SCD stroke risk screening. Flow velocities were derived from pulsed-wave spectral Doppler waveforms; colour flow imaging was not used for velocity quantification. Participants were assessed lying down in a quiet room, with a caregiver present for younger children. Insonation was performed bilaterally through the trans-temporal window, targeting the middle cerebral artery (MCA). As non-imaging TCD was used, vessel identity was inferred from insonation depth, flow direction, and tracking to the internal carotid artery (ICA) bifurcation rather than direct visualization. The MCA was identified at depths of 34–40 mm and followed proximally to the ICA bifurcation to confirm vessel identity. Gain and filter settings were adjusted as needed to obtain a clean waveform, and at least three stable consecutive waveforms were recorded per side. Where the temporal window was poor on one side, this was documented and the contralateral measurement was used.

The primary velocity parameter was the MCA mean peak flow velocity. Where both sides were successfully recorded, the higher of the two values was used for velocity categorization, consistent with standard practice in paediatric SCD screening.

Data from both arms were entered into a secure, access restricted REDCap database on a password protected server. Identifiers were replaced with unique study codes, and data validation checks were performed to resolve missing, inconsistent, or outlier entries before export of the de-identified dataset for analysis.

**TCD Velocity Classification**

Cerebral blood flow velocities were classified according to the criteria described in a Tanzanian TCD study of children with sickle cell disease (12). This classification system was selected because it was validated in a Tanzanian pediatric population with characteristics similar to our study population.

The velocity categories were: Low: <50 cm/s, normal: 50–149 cm/s; slightly elevated: 150–169 cm/s, conditional risk: 170–199 cm/s and abnormal: ≥200 cm/s. TCD risk categories were based on the middle cerebral artery mean peak flow velocity (cm/s).

**S4: Data analysis**

Data analysis was conducted using both descriptive and inferential methods using Stata version 17. Demographic and clinical characteristics were summarized using frequencies and percentages for categorical variables, including gender, ethnicity, prior admission, use of hydroxyurea, history of blood transfusion, and developmental delay. Continuous data such as age and weight were summarized using median and range. Haemoglobin level was analysed descriptively using frequencies and percentages. Haemoglobin level of (<10 g/dl) was considered low.

The prevalence of abnormal cerebrovascular flow velocity (CBFV) measurements was determined as a proportion of the total sample size. This was based on TCD values, which were categorized into low (< 50 cm/s), Normal (50-149 cm/s), slightly elevated (150- 169 cm/s), conditional risk (170-199 cm/s), and abnormal (≥ 200 cm/s). Velocity categories were assigned using the middle cerebral artery mean peak flow velocity (cm/s) recorded during TCD assessment. The cerebral blood flow velocity (CBFV) measurements, including depth, peak systolic velocity, end diastolic velocity, and mean peak flow velocity, were summarized descriptively using median and range.

Fisher’s exact test was performed to compare the demographic, clinical characteristics, and hemoglobin levels across TCD velocity categories.

A Wilcoxon rank-sum test was performed to assess differences between the left and right recordings based on age. The test compared the distributions of values across the two sides to assess whether the median ranks differed significantly. Significance was considered at 5%.

Given the small sample size in the cross-sectional arm, analyses for children without SCD were primarily descriptive.

**S5: Ethical considerations**

Ethical approval for this study was obtained from the Research Ethics Committee of Gertrude’s Children’s Hospital and the Aga Khan University, Nairobi Institutional Scientific and Ethics Review Committee (ISERC). Permission for data collection and use was obtained from Ruaraka Uhai Neema Hospital to ensure compliance with institutional and ethical research guidelines. Additionally, a research permit was obtained from the National Commission for Science, Technology, and Innovation (NACOSTI), in accordance with national research regulations. This study adhered to the International Council for Harmonization (ICH) Good Clinical Practice (GCP) Guidelines.

A waiver of consent was granted by ISERC, as the research involved the analysis of existing data from a routine clinical procedure with no additional new interventions. The study posed minimal risk to participants. Patient confidentiality was maintained by de-identifying clinical data, which was stored in a password-protected database accessible only to the study team.

Written informed consent was obtained from parents/guardians prior to enrollment in the cross-sectional arm, and assent was obtained from children aged 7 years and above.

**Supplementary Appendix: List of Abbreviations**

ACA, anterior cerebral artery; CBFV, cerebral blood flow velocity; Ex-ICA, extracranial internal carotid artery; GCH, Gertrudes Children’s Hospital; GCP, Good Clinical Practice; Hb, hemoglobin; HbSS, hemoglobin SS (sickle cell anemia); HU, hydroxyurea; ICU, intensive care unit; IQR, interquartile range; ISERC, Institutional Scientific and Ethics Review Committee; MCA, middle cerebral artery; NACOSTI, National Commission for Science, Technology, and Innovation; PCA, posterior cerebral artery; RBC, red blood cell; REDCap, Research Electronic Data Capture; RUNH, Ruaraka Uhai Neema Hospital; SCA, sickle cell anemia; SCD, sickle cell disease; SSA, Sub-Saharan Africa; STATA/Stata, statistical software used for analysis; STOP, Stroke Prevention Trial in Sickle Cell Anemia; TAMMV, time-averaged mean of maximum velocity; TCD, transcranial Doppler; WHO, World Health Organization.
